# Preoperative CT-based detection of extrapancreatic perineural invasion in pancreatic cancer

**DOI:** 10.1038/s41598-021-81322-4

**Published:** 2021-01-19

**Authors:** Ekaterina Khristenko, Igor Shrainer, Galia Setdikova, Oxana Palkina, Valentin Sinitsyn, Vladimir Lyadov

**Affiliations:** 1 Department of Diagnostical and Interventional Radiology, Heidelberg University Hospital, Heidelberg, Germany; 2 Department of Radiology, First Moscow City Hospital, Moscow, Russia; 3 Department of Pathology, Botkin Moscow City Hospital, Moscow, Russia; 4 Department of Radiology at Medical Educational and Scientific Center University Hospital, Lomonosov Moscow State University, Moscow, Russia; 5 Department of Oncology N4, City Clinical Cancer Hospital N1, Moscow, Russia; 6 Chair of Oncology and Palliative Medicine, Russian Medical Academy of Postgraduate Professional Education, Moscow, Russia

**Keywords:** Cancer imaging, Medical research, Oncology, Signs and symptoms, Cancer, Gastrointestinal diseases

## Abstract

Accuracy for computed tomography (CT) diagnosis of extrapancreatic perineural invasion (EPNI) in pancreatic ductal adenocarcinoma (PDAC), which is a significant cause of recurrence, has not been established. The aim of the study was to evaluate the diagnostic accuracy of CT in detecting EPNI preoperatively in resectable PDAC of the pancreatic head. Retrospective study design was approved by institutional review board. Preoperative CT-series of 46 patients with resectable PDAC were evaluated by two independent observers. Plexus Pancreaticus Capitalis-II (PPC-II) was assessed as this area is more susceptible for EPNI. All patients underwent surgery with dedicated histopathology, which served as the reference standard. Histologically EPNI was confirmed in 63.1%. Sensitivity of MDCT was 93.1% (95% confidence interval (CI) 77.23% to 99.15%), specificity 64.7% (95% CI 38.33% to 85.79%) with area under the curve (AUC) 0.789 for the first observer. Positive predictive value (PPV) was 81.82% (95% CI 70.12% to 89.62%), negative predictive value (NPV—84.62% (95% CI 57.98% to 95.64%) with diagnostic accuracy of 82.61% (95% CI 68.58% to 92.18%). Interobserver agreement showed k-value of 0.893 (${p} < 0.001$), which represents very good agreement between observers. Median actual survival in patients without EPNI was 30 months (95% CI 18.284–41.716), in patients with EPNI—13 months (95% CI 12.115–13.885). CT provides sufficient diagnostic information to detect PPC-II invasion in patients with resectable PDAC of the pancreatic head. Preoperative detection of EPNI might be an additional argument to perform neoadjuvant chemotherapy in patients with resectable PDAC. It should be included in preoperative evaluation form of CT-findings.

## Introduction

PDAC remains one of the deadliest cancers despite recent advances in imaging, surgery, and chemotherapy. It has one of the lowest 5-year survival rates compared to other solid cancers regardless of the stage of the disease (10%)^1^ and one of the reasons is that more than half of the patients are diagnosed with a widespread metastatic disease.

Surgery is the major treatment modality providing a chance for cure in pancreatic cancer patients. However, reported perioperative mortality and morbidity rates for pancreatoduodenectomy approach 5% and 60%, respectively. Moreover, according to some authors even in patients with resectable stage I-II tumors 5-year survival rate reaches only 20%, which led to the adoption of adjuvant chemotherapy as a mainstay treatment^2,3^. Recently, Conroy et al.^4^ have shown in a randomized trial that a selected cohort of resected patients might benefit from an aggressive adjuvant chemotherapy regimen FOLFIRINOX with a median overall survival of 54 months.

An important problem is the high rate of locoregional recurrences after seemingly “radical” surgical procedures. Several authors have shown that a standardized specimen examination protocol with a redefined R1 definition (tumor in < 1 mm instead of 0 mm margin) reveals R1 resection status in as much as 70% or more of pancreatic resections^5,6^.

The most vulnerable margins are shown to be mesenteric (medial) and posterior usually due to perineural invasion along the plexus surrounding superior mesenteric artery (SMA). This is perhaps not surprising as far as extrapancreatic perineural invasion represents a distinct route of spread in PDAC which is sometimes found even in tumors less than 2 cm in size^7^. Until recently both intrapancreatic and extrapancreatic perineural spread were diagnosed only histopathologically. However, the wide adoption of ultrathin CT protocols led to the recognition of EPNI among radiologists^8,9^.

Extrapancreatic neural plexuses include six main pathways: celiac, SMA, pancreatic head, hepatic, aortic, and splenic plexuses. Non-affected thin neural bundles are not visible on CT-scans. Yet larger neural structures such as celiac and SMA ganglia can be visible on CT as small linear structures on the level of Th12-L1^10,11^. The most important pathways of extrapancreatic perineural invasion in pancreatic head cancer are plexus pancreaticus capitalis I (PPC I), plexus pancreaticus capitalis II (PPC II) and gastroduodenal artery plexus (which is also known as an anterior pathway)^7^. PPC I and PPC II were first described as the major neural pathways in the pancreatic head region in a cadaveric study by Yoshioka and co-authors in 1985^11^.

There are several prior reports in the literature on correlation between radiological findings and histopathological detection of EPNI^7–9,12,13^. Yet, clear CT-signs of SMA, celiac axis or hepatic artery encasement make the search for EPNI clinically irrelevant due to unresectability. It seems that the most intriguing question would be if preoperative EPNI diagnosis might be a major predictor of poor prognosis in resectable or borderline resectable tumors. Therefore, the aim of the present study was to find whether CT can detect EPNI in resectable adenocarcinoma of the pancreatic head with a good accuracy and reproducibility. To underline the importance of preoperative EPNI detection in patients with otherwise resectable cancer, we combined CT evaluation of EPNI with dedicated histopathological specimen evaluation and survival analysis.

## Results

### Baseline results

The mean age of the patients was 57.2 ± 18.7 years. Obstructive jaundice was present in 37 of 46 patients at initial presentation (80%) and 26 patients (57%) had some type of preoperative biliary drainage before radical surgery. In 9 patients without jaundice tumor was found because of epigastric pain or as an incidental finding. All the patients underwent pancreatoduodenectomy with a meticulous excision of the right half of the SMA neural plexus as well as peripancreatic lymph node dissection. In 10 out of 46 patients (22%) a segmental resection of superior mesenteric vein (SMV) was performed. There were no arterial resections in this group of patients.

### Pathohistological results

Pathologically two types of perineural invasion were identified: intrapancreatic and extrapancreatic. It was not possible to differentiate intrapancreatic invasion on CT scans. The presence of EPNI was histologically confirmed in 29 patients (63.1%), 23 of those patients (79.3%) also showed intrapancreatic perineural invasion.

In the group with histologically verified EPNI there was one pT1N1M0 patient, one—pT2N0M0, 2—pT2N1M0, 8—pT3N0M0 and 17 pT3N1M0 patients. In the group without EPNI there were 3 pT2N0M0 patients, one—pT2N1M0, 4—pT3N0M0 and 9 pT3N1M0 patients.

### Imaging findings

**Figure 1:**
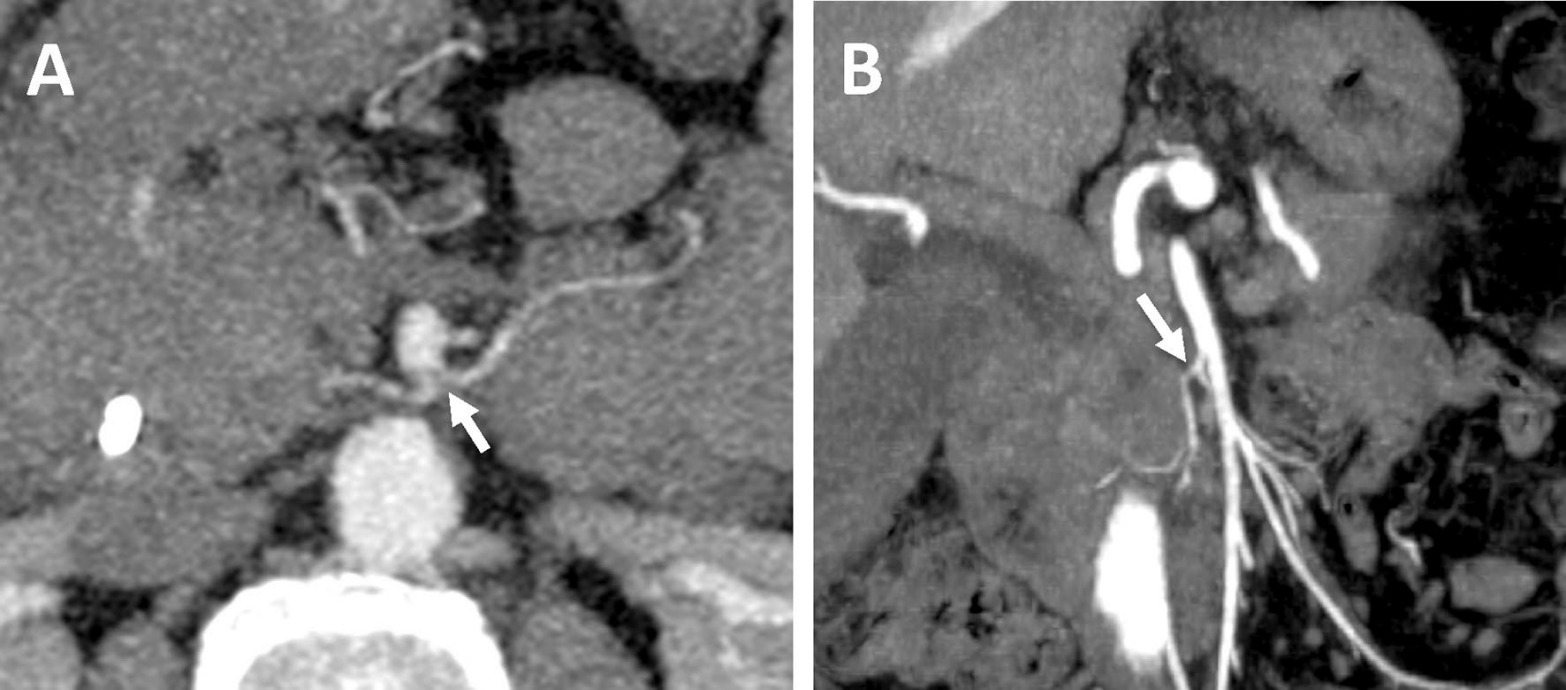
CT-examinations of two different patients with adenocarcinoma of pancreatic head obtained with intravenous contrast material. (**A**) Axial MIP-image in arterial phase shows the common origin of inferior posterior pancreatoduodenal artery and first jejunal branch (arrow), arising from superior mesenteric artery (**B**) oblique coronal MIP-image shows inferior pancreatoduodenal artery and its branches with its common origin with the first jejunal branch (arrow).

Primary tumor was visualized on CT images as a hypodense lesion with ill-defined margins and a hypovascular pattern of contrast enhancement. The mean size of the tumor was 25.1 ± 6 mm. The majority of the tumors were homogenous, one showed calcification and two tumors had a necrotic component. The mean HU-density in the late arterial phase was 61.5 + 5.5 HU with density of adjacent pancreatic parenchyma of 138.5 + 6.5 HU. Abutment of arterial vessels was present in 14 patients. Encasement or abutment of SMV in 13 patients, 10 of those subsequently underwent SMV-resection. Detailed CT findings according to our assessment protocol are listed in Table 1. We focused on the assessment of PPC II as the most common way of tumor spread in the pancreatic head cancer with the majority of tumors exhibiting extrapancreatic growth towards the superior mesenteric artery along the inferior pancreatoduodenal artery (IPDA). The IPDA was visualized in all patients. In the majority of cases it was the first branch of the SMA having its own origin; in other cases, it had a common origin with the first jejunal artery (Fig. 1).

**Table 1:** Preoperative CT characteristics of 46 patients with adenocarcinoma of pancreatic head (Observer 1).

CT assessment protocol ($\hbox {n}=46$)	
Tumor size (mm)	25.1 ± 6
**Tumor structure (n)**	
Homogenous	43 (93.5%)
Calcifications	1 (2.2%)
Zones of necrosis	2 (4.3%)
Density of normal pancreatic parenchyma in late arterial phase (HU)	138.5 ± 6.5
Tumor density in late arterial phase (HU)	61.5 ± 5.5
Intraluminal duct density (HU)	18 ± 6
Density of infiltrated fat tissue in native phase (HU)	10 ± 8
**Duct obstruction (n)**	
Common hepatic duct (> 10 mm)	12 (26%)
Main pancreatic duct (> 3 mm)	14 (30%)
Common bile duct	15 (32.6%)
No obstruction	5 (10.9%)
**Signs of infiltration along arterial vessels (n)**	
Celiac trunk (< 1800)	2 (4.3%)
Hepatic artery (< 1800)	4 (8.7%)
Superior mesenteric artery (< 1800)	8 (17.4%)
Inferior pancreatoduodenal artery	33 (71.7%)
**Signs of infiltration along venous vessels (n)**	
Portal vein (< 1800)	4 (8.7%)
Superior mesenteric vein (< 1800)	8 (17.4%)
Superior mesenteric vein (> 1800)	13 (28.2%)
Confluence	4 (8.7%)
Infiltration of duodenal wall (n)	31 (67.4%)
Liver metastasis (n)	0
Locoregional lymphadenopathy (n)	23 (50%)

**Figure 2:**
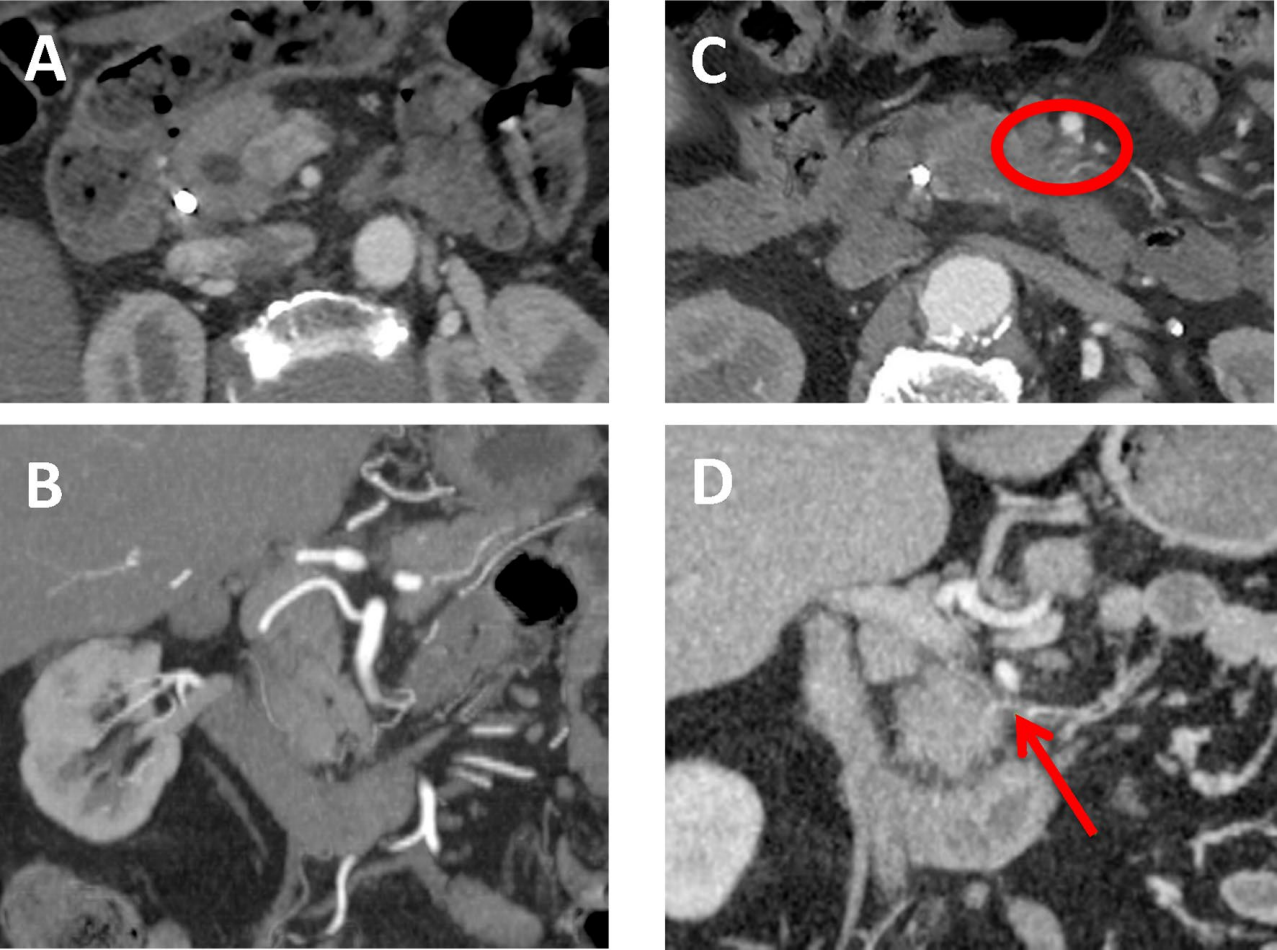
CT-examinations of two different patients without CT-signs of perineural invasion (**A**,**B**) and with CT-signs of perineural invasion (**C**,**D**) obtained with intravenous contrast material. Axial MIP scans (**A**) and oblique MIP coronal reformat (**B**) in arterial phase demonstrate normal fatty tissue surrounding IPDA which suggests the absence of perineural spread. Axial MIP scans (**C**) and oblique coronal reformat (**D**) in arterial phase demonstrate the areas with soft tissue attenuation surrounding IPDA (red circle and red arrow), reported as suspicious for perineural invasion.

The main clue for assessment of the perineural invasion in the pancreatic head region was visualization of normal fatty tissue surrounding the IPDA on both axial scans and coronal reformats (Fig. 2)

We compared CT findings with pathological results for both observers (Table 2). 27/29 patients with positive PPC II invasion were correctly diagnosed on preoperative CT-scans by observer one and 26/29 by observer two. Sensitivity of MDCT was 93.1% (95% CI 77.23–99.15%), specificity 64.7% (95% CI 38.33–85.79%) with AUC 0.789 for the first observer and sensitivity 89.7 % (95% CI 72.65–97.81%), specificity 58.8% (95% CI 32.92–81.56%) with area under the curve (AUC) 0.742 for the second observer. Positive predictive value was 81.82% (95% CI 70.12–89.62%), negative predictive value 84.62% (95% CI 57.98–95.64%) with a diagnostic accuracy of 82.61% (95% CI 68.58–92.18%) for observer one and positive predictive value was 78.8% (95% CI 67.50–86.92%), negative predictive value 76.9% (95% CI 51.53–91.27%) with a diagnostic accuracy of 78.3% (95% CI 63.64–89.05%) for observer two. Employing a crosstab interobserver agreement showed a Kappa-value of 0.893 with a standard error of 0.074 (${p} = 0.000$), which represents very good agreement between two observers and suggests good reproducibility of the results.

**Table 2:** Comparison of CT findings of extrapancreatic perineural invasion with pathological results in 46 patients and the ROC-curves for both observers.

	Pathology		Total
	Negative	Positive	
**Infiltration assessment—Observer 1**			
CT			
Negative	11	2	13
Positive	6	27	33
Total	17	29	46
**Infiltration assessment—Observer 2**			
CT			
Negative	10	3	13
Positive	7	26	33
Total	17	29	46

**Figure 3:**
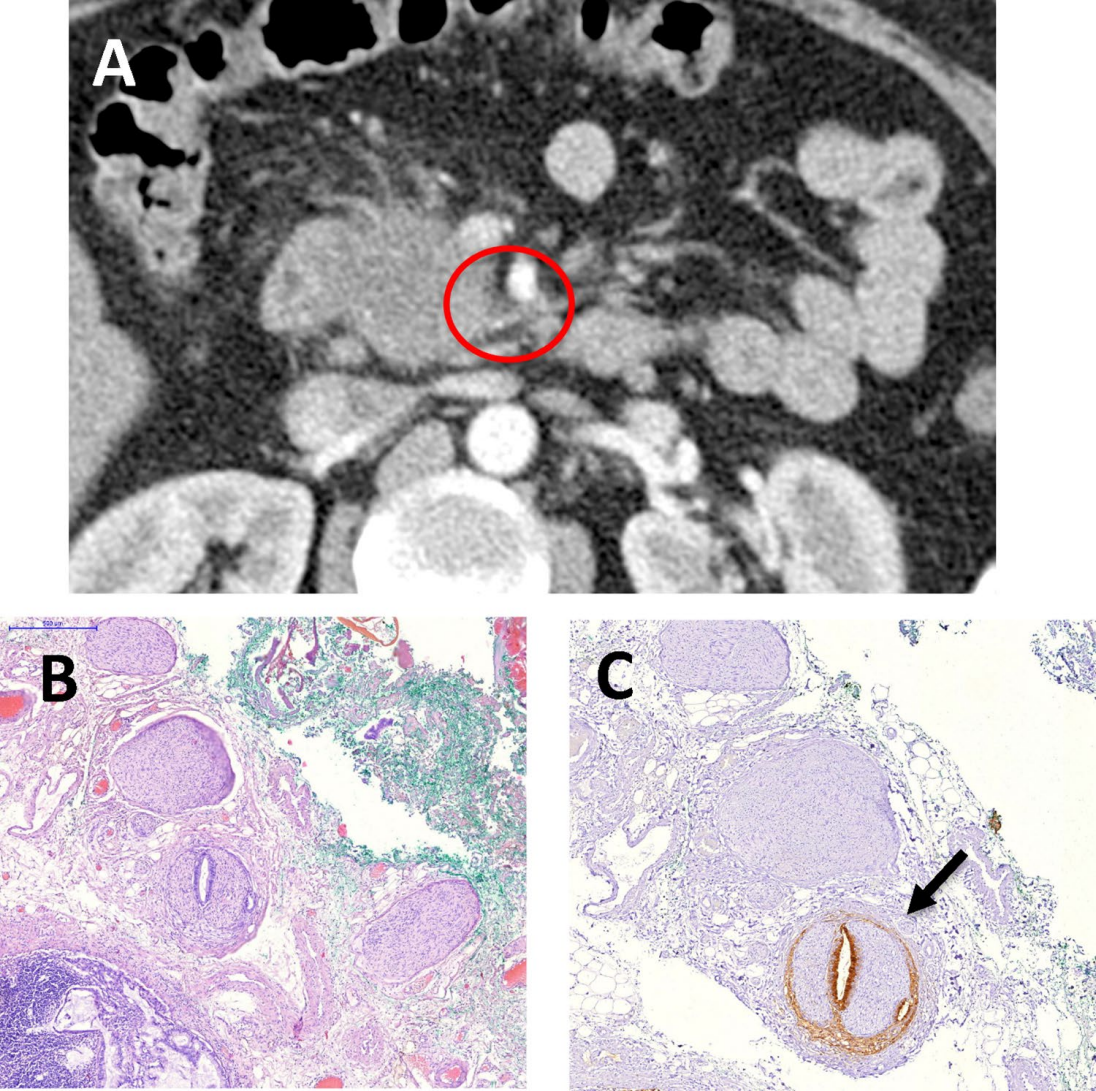
CT-examination obtained with intravenous contrast material (**A**) and microscopic specimen (**B**,**C**) of a 64 years old male patient with adenocarcinoma of pancreatic head. (**A**) Axial CT scan in arterial phase reveals the area of soft tissue attenuation along the course of IPDA. There is a contact to SMV < 180 with no signs of infiltration of SMA, so the primary tumor was evaluated as resectable. (**B**,**C**) The pathological section on the corresponding level to CT scan with hematoxylin and eosin staining. (**B**) The medial resection margin is inked with green stain. (**C**) Immunohistochemistry examination shows an expression of mucin Typ I in the tumor cells, which is marked with brown staining (arrow), demonstrating extrapancreatic perineural invasion.

An example of a CT-study with true positive result, verified histopathologically, is shown in Fig. 3.

**Figure 4:**
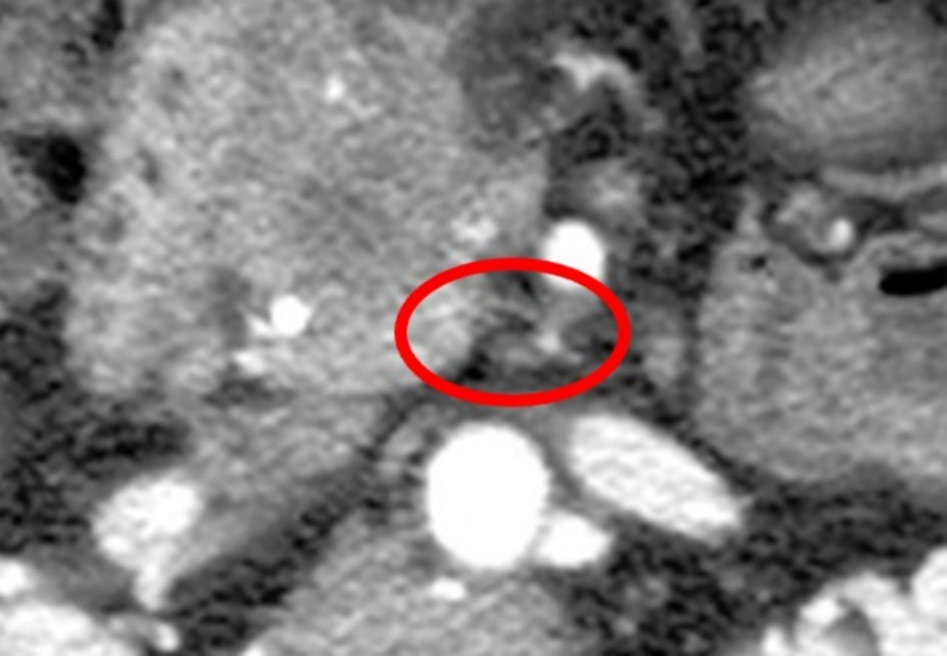
Axial CT scan in arterial phase shows Pattern 2 of EPNI, that represents infiltration along the IPDA in the reticular pattern without tumor contact.

In 13 patients (28.2%) there were no signs of infiltration along the IPDA. In 33 cases (71.7%) there were signs of infiltration along the IPDA, showing different patterns. It was depicted as an increased density along the IPDA either having contact with the tumor or without continuous spread. Thus, we identified two patterns of perineural spread: pattern 1 was identified as a soft tissue infiltration with delineated contours and tumor contact (already shown in Fig. 2, Image C) and pattern 2 as perivascular infiltration without continuity to the tumor (Fig. 4). It was easier to interpret Pattern 1 changes than Pattern 2.

In 33 patients with positive EPNI according to CT findings Pattern 1 was found in 21 patients (63.6%) and Pattern 2 was observed in 12 patients (36.4%). According to that we performed a further correlation with pathological findings and comparison between these two patterns. In the group with Pattern 1 EPNI was present in 95.24% of patients and in the group with Pattern 2 EPNI was present in 45.15%. Employing Mann Whitney U test the difference between these two groups was statistically significant (${p} = 0.003$). The majority of false positive results were associated with Pattern 2 (6 out of 7 cases, 85.71%). All false positive patients underwent R0 resection. In Pattern 1 the tumor stage was T2N0M0 and in Pattern 2 there were 4 patients with T3N1M0 and 2 patients with T3N0M0. Histologically they were reported as fibrosis (4) which is a common feature of pancreatic adenocarcinoma or inflammatory changes (3) that might be caused by tumor-induced pancreatitis.

EPNI was more often encountered in patients with more advanced tumors according to their pathological stage. In patients with histologically proven EPNI pT3 stage dominated with the rate of 86.20% (among them pT3 pN0 pM0 in 8 patients and pT3 pN1 pM0 in 17 patients). In comparison to that in the group without EPNI pT3 stage was found in 76.47% (among them pT3 pN0 pM0 in 4 patients and pT3 pN1 pM0 in 9 patients), there was no statistically significant difference between the groups.

**Figure 5:**
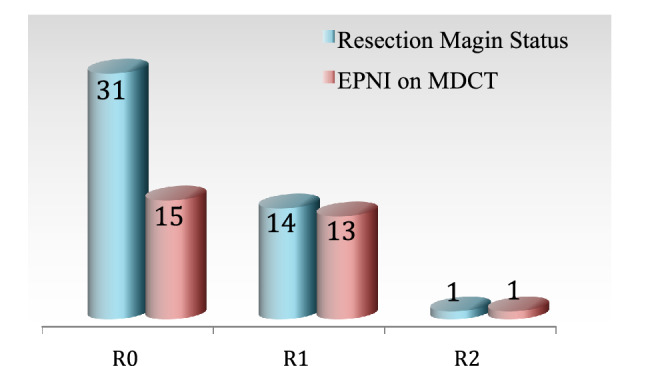
Comparison of CT findings of extrapancreatic perineural invasion with R-status.

There were 31 R0 resections, 14 R1 resections and 1 R2 resection. In the patient with macroscopically not radical (R2) resection both perineural invasion of PPC II and PPC I was found. We found CT signs of perineural invasion in 15 out of 31 patients with R0 resection, in 13 out of 14 with R1 resection and also in the only one with R2 resection (Fig. 5).

**Figure 6:**
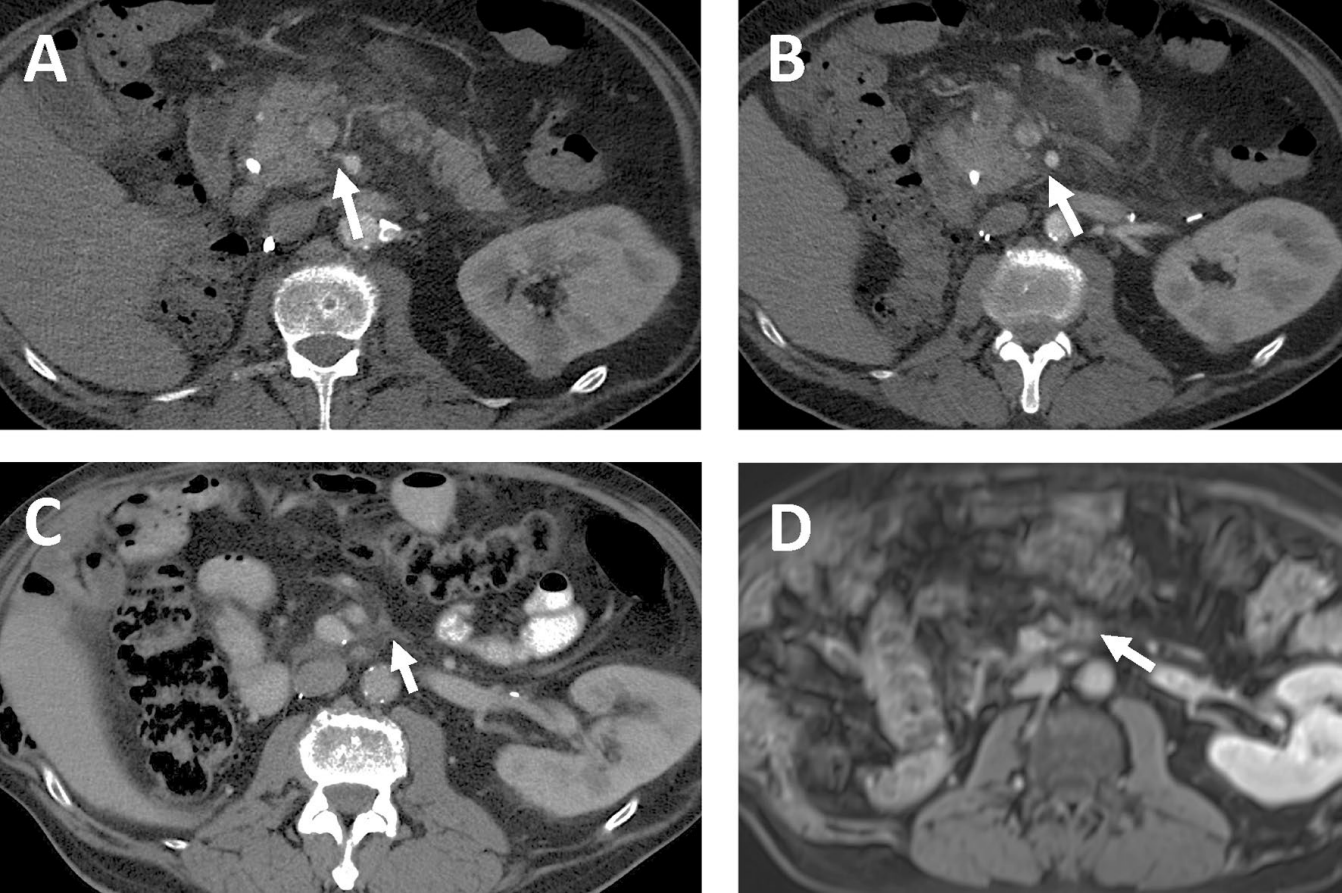
Patient with histologically confirmed pT1c, pN1 (1/14), L0, V0, R0 G2 tumor with positive perineural invasion in the dorsal area of resection. (**A**,**B**) Both axial images in the arterial phase. Origin of IPDA arising from SMA with subtle diffuse infiltration along the IPDA and in the peripancreatic tissue (arrows), reported as Pattern 2 by the first observer and negative by the second observer. (**C**) First postoperative CT study within 3 months after resection showing areas of focal increased attenuation left to SMA (arrow). (**D**) Second postoperative study within 5 months after resection (MRI was performed to verify unclear liver finding within the previous CT study). Focal area of soft tissue intensity and enhancement (arrow) has increased in size and was interpreted as local recurrence.

A clinical example of a patient who underwent an R0 pylorus-preserving pancreatoduodenectomy and subsequent 6 cycles of adjuvant chemotherapy with Gemcitabine is shown in Fig. 6. This patient developed a local recurrence of adenocarcinoma within 9 months after radical R0 resection. Preoperatively his CT-study demonstrated Pattern 2 EPNI reported by the first observer and was EPNI-negative in the opinion of the second observer (false negative). Histologically there was a pT1c tumor with positive perineural invasion in the dorsal area of resection with a 6 mm distance to the marked dorsal margin.

**Figure 7:**
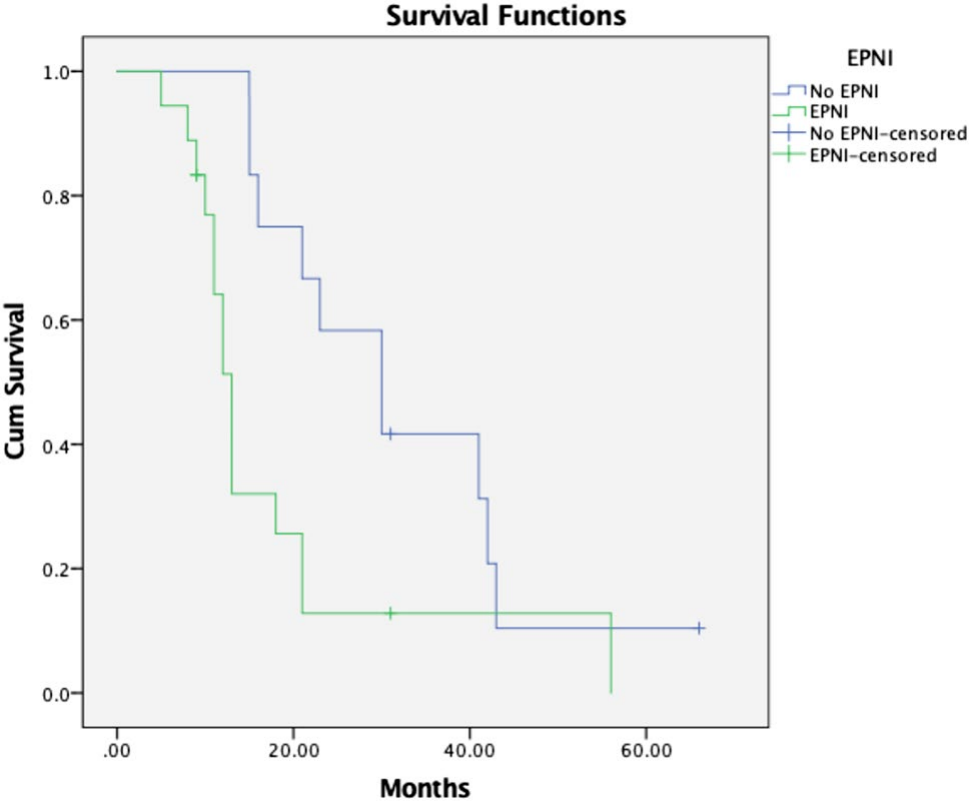
Kaplan-Meier survival analysis in patients with PDAC with and without EPNI.

We performed a survival analysis in our group of patients. The results of 8 patients were excluded because of in-hospital or 90-days postoperative mortality, thus we found it inappropriate to include them in the survival analysis. According to Kaplan-Meier survival analysis (Fig. 7) the median actual survival rate in patients without EPNI was 30 months (95% CI 18.284–41.716) and in those with EPNI—13 months (95% CI 11.564–14.436), (${p} = 0.073$).

Using a Cox regression analysis, a presence of EPNI increased a risk of both death and progression showing hazard ratio for death of 1.987 (95% CI 0.776–5.008) and for progression—1.992 (95% CI 0.778–5.097), but didn’t reach the statistical significance.

## Discussion

In our study we combined CT evaluation of EPNI, a dedicated histopathological specimen evaluation and survival analysis to underline the importance of preoperative EPNI detection in patients with otherwise resectable cancer of the pancreatic head. Also, we observed 2 distinct patterns of EPNI along the SMA according to CT, which appeared to be a highly reproducible way ($\hbox {k} = 0.893, {p} = 0.000$) to evaluate EPNI before surgery. Our study showed that CT-based diagnosis of pattern 1 EPNI (continuous spread of tumor along the IPDA) has a high level of correlation with histopathological findings (95.24%). Its value as a prognostic factor should be further evaluated in prospective trials.

In concordance with the literature, radiologically EPNI was identified as confluent tissue of similar attenuation to primary pancreatic cancer extending along the known course of PPC II^12,13^. It was highly prevalent in our group of patients with PDAC (63.1%), which is comparable with previously reported rates over 70%^13,14^. In our study the presence of EPNI was histologically confirmed even in one patient with pT1c and 3 patients with pT2 stage, which corresponds to the reported findings of EPNI in patients with small PDAC^8^.

Our study supported the finding that PDAC of the pancreatic head most often spreads in the direction of SMA, which makes the area along the IPDA and PPC II important for the detection of extrapancreatic spread / perineural invasion. According to our findings, the presence of EPNI might be overestimated by radiologists in Pattern 2 cases, so the most precise attention should be paid to cases when infiltration along the IPDA is present without continuous extension to the primary tumor. The absence of continuous spread of the tumor along the arteries leads to a significant limitation in interpreting EPNI and differentiation of EPNI and desmoplastic reaction. Radiologically it was not possible to differentiate fibrotic changes from infiltration (neoplastic or inflammatory) in Pattern 2.

Preoperative detection of EPNI, which is reported to be a significant cause of positive surgical margins and tumor recurrence, could influence the clinical management of patients in terms of performing surgical treatment or neoadjuvant therapy first. Neoadjuvant therapy in pancreatic cancer is an option which gains more and more credit. Yet, no definitive answer exists regarding its value and indications in patients with apparently resectable PDAC. Our finding of low postoperative survival in patients with histologically proven EPNI supports our opinion that the detection of different types and patterns of EPNI and precise assessment of arterial vessels involvement is crucial for clinical decision making. Radiological assessment of EPNI can potentially allow an early detection of patients with aggressive disease and develop an additional indication for neoadjuvant chemo- or chemoradiation therapy in patients with otherwise resectable disease.

Recently Zins et al. underlined that the limitation of PDAC staging in the era of neoadjuvant therapy is the absence of routine preoperative perineural invasion assessment by imaging^15^. They also suggest that in some patients with seemingly R0 resection the presence of occult perineural invasion might explain the occurrence of rapid systemic spread or locoregional treatment failure. Current radiological criteria do not include the assessment of EPNI^16,17^. We believe that in the shown case (Fig. 6) with local recurrence in R0 resection the cause of the perivascular recurrence was the presence of perineural invasion in the dorsal area of resection, although the tumor stage was pT1c, pN1 (1/14), L0, V0, R0 G2.

Perineural invasion is known to be a major determinant of positive surgical margins and recurrence after pancreaticoduodenectomy^18^. In our study we presented an example of the development of early locoregional recurrence in a patient with R0 resection and histologically proven EPNI. Precise evaluation of perivascular changes is important not only during the assessment of primary tumor spread but also during follow-up studies, especially after successful radical pancreaticoduodenal resections. While scrutinizing tomograms for revealing the local tumor recurrence, potential sites of extrapancreatic perineural invasion should be assessed.

It was reported that perivascular tissue more than 2 mm in width was associated with perineural invasion in more than 90% of patients with PDAC^19^. Those subtle changes are a cause of potential false positive assessment and should be differentiated from small lymphatic nodes and obliterated inferior posterior pancreatoduodenal vein (IPDV), which is a challenging task for observers. There is a potential bias in interpreting changes of IPDV, which runs along IPDA. IPDV may be depicted as soft tissue attenuation along IPDA in the arterial phase, and it should be ruled out at the second phase. One should pay attention to the obliteration of IPDV and dilatation of other PDVs, as this finding was reported to be an important sign of EPNI^20,21^. However, anatomically IPDV and IPDA lie in slightly different layers with neural bundles predominantly encasing the artery. Therefore, IPDV assessment was not the focus of our research.

According to our findings CT doesn’t allow to differentiate intrapancreatic invasion and microscopic extrapancreatic invasion. CT has quite a high sensitivity, but moderate specificity in the detection of EPNI. Other changes such as fibrosis and inflammation could look similar to perineural invasion, making increased density or soft tissue appearance around IPDA an unspecific finding.

There are several limitations to our study. First major limitation is the limited number of patients, partly explained by the exclusion of the patients who underwent neoadjuvant chemotherapy. It was important as post-therapeutic changes can be undistinguishable from the true perineural invasion on CT scans. Also, we didn’t include the results of PPC I assessment as far as its involvement is a very infrequent situation compared to the PPC II invasion. The second major limitation is the retrospective study design, which can be partly explained by the absence of EPNI assessment in the current clinical guidelines.

## Conclusion

Our findings show that CT is capable to detect the presence of EPNI preoperatively in patients with previously untreated PDAC. However, CT demonstrates some false positive results, especially in cases when there is no visible contact between the tumor itself and the changes along the IPDA. In the survival analysis we found that median actual survival rate in patients without EPNI was more than twice longer than in patients with EPNI. This finding underlines the importance of CT-based preoperative evaluation of EPNI regarding the management of resectable pancreatic cancer. We suggest including these criteria in preoperative assessment and reporting, although current radiological criteria do not include the assessment of EPNI. CT presentation of EPNI appears to be essential for radiologists and clinicians as far as it helps to identify patients with more aggressive disease.

## Methods

### Ethics approval and consent

This study was performed according to the Declaration of Helsinki. Ethical approval was obtained from the local ethical review board of Russian Medical Academy of Continuous Postgraduate Education (RMANPO, Number 8, 21.10.2014). Due to its retrospective design, the need for an informed consent was waived by the Review board.

### Patient selection and data collection

The database of the Center of Medicine and Rehabilitation (Moscow, Russia) was searched retrospectively for the patients who underwent surgical resection of previously untreated PDAC between March 2009 and April 2014 and who had available CT examinations not more than 1 month before surgery. Clinical data and histopathological reports of those patients were retrieved from the hospital information system. All the patients underwent pancreatoduodenectomy with a meticulous excision of the right half of the SMA neural plexus as well as peripancreatic lymph node dissection.

Data such as age, gender, symptoms, laboratory tests, histopathological findings, follow-up, and imaging findings were retrospectively analyzed. The exclusion criteria included neoadjuvant therapy before surgery and non PDAC histology. We identified 46 patients (21 male and 25 female) with clinical and radiological signs of resectable / borderline resectable solid mass in the pancreatic head, suspicious for PDAC. The tumor, node and metastasis (TNM) staging system used in the study was the 7th edition of AJCC (American Joint Committee on Cancer) system (2009).

### CT scans

Multiphase CT-scans were obtained no longer than one month before surgery with a slice thickness of 1.5 mm. CT-studies were performed on Somatom Sensation 64 MDCT scanner (Siemens) and Discovery 750 DECT (GE Healthcare) with intravenous injection of a contrast medium. Studies included unenhanced phase, late arterial phase (40 seconds after the bolus injection of contrast medium) and portal venous phase (60-70 seconds after the bolus injection of contrast medium). Contrast medium was injected in the cubital vein using an automatic injector with the mean volume of 100 ml and injection rate of 3 ml/second. Nonionic contrasts were used with iodine concentration of 350 mg/ml (Omnipaque, GE Healthcare). Technical parameters for the studies included a tube voltage of 120 kV and a tube current of 170 mA (effective). Scanning was performed with a breath hold of 9-12 seconds. The radiation dose varied from 11 to 25 mSv. For postprocessing, workstations «Leonardo »(Siemens) and «Windows Advantage 4.4 and 4.5 »(GE) were used. Additionally, the assessment of multiplanar reconstructions (MPR) in the arterial and portal venous phase was performed, as well as volume rendering (VR) and maximum intensity projections (MIP) reconstructions.

### Image analysis

Retrospective evaluation of MDCT examinations was made by two independent radiologists (EAK with 5 years of experience in preoperative staging of PDAC and IVS—10 years). Both contrast-enhanced data sets (late arterial and portal venous) were assessed using MPR, MIP, and VR postprocessing. No additional postprocessing software was employed. Both observers were blinded to the results of the histopathological reports.

The readers analyzed the local extension of PDAC, including tumor size, tumor structure, tumor densities in the unenhanced and late arterial phases, density of pancreatic parenchyma in the late arterial phase, intraluminal duct density, duct obstruction, infiltration along the arterial structures, such as celiac trunk, hepatic artery (HA), superior mesenteric artery (SMA) and inferior pancreatoduodenal artery (IPDA) and along the venous structures such as portal vein (PV), superior mesenteric vein (SMV) and confluence, infiltration of duodenal wall, liver metastasis, and the presence of lymphadenopathy.

According to the aims of our study, precise analysis of tumor invasion along the PPC II, namely along the neural plexuses around SMA, IPDA and jejunal trunk, was made. EPNI was identified as confluent tissue of similar attenuation to primary pancreatic cancer extending along the known course of inferior pancreatoduodenal artery.

### Pathological evaluation

Histopathology served as the reference standard in this diagnostic accuracy study. According to Royal College of Pathologists guidelines for pancreatoduodenectomy specimen reporting R1 status was defined as the presence of tumor tissue $\le 1$ mm from a circumferential margin surface^22^.

**Figure 8:**
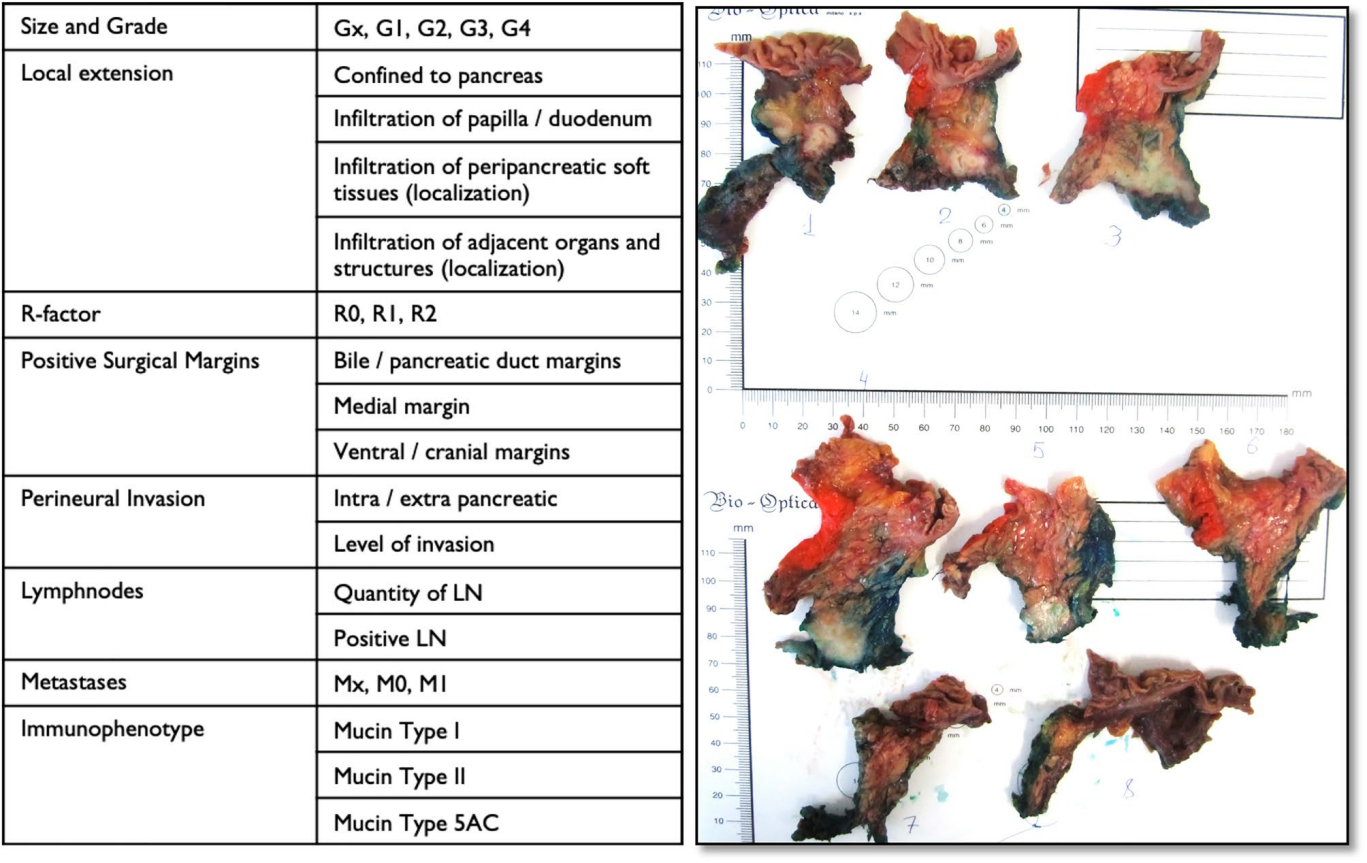
An example of our dedicated histopathological protocol with a specimen after inking. The specimen of a resected ductal adenocarcinoma of the pancreatic head (G2) with intrapancreatic perineural invasion (pT3) of a gastric type (Mucin Type 5AC expression). The tumor is located less than 1 mm from the medial resection margin and infiltrates the parapancreatic tissues (R1). Stage IIB, pT3 pN1 cM0. Slice thickness of the specimen 0.5 cm, EPNI was reported to be present in the first 4 slices.

A standardized histopathological protocol with intraoperative marking and assessment of surgical margins was used with a special focus on surgically marked SMA margin. Immediately after pancreatoduodenectomy the SMA margin was separately marked with a string for a detailed histopathological evaluation which was a part of our standard specimen handling protocol. The surgical margins were inked as follows: medial resection surface with green ink, dorsal—blue, ventral—orange. The pathological assessment was carried out by two dedicated pancreatic pathologists (GRS, OVP). During this assessment the pathological specimens were cut into 0.5 cm transaxial slices, identical to transversal CT scans. The level of detected EPNI was provided in the dedicated protocol in order to make a more precise correlation with the axial CT images. Depending on the size of specimen there was a different number of slices pro patient, with a mean value of 10. An example of our dedicated histopathological protocol is shown Fig. 8.

### Statistical analysis

Data was recorded using a spreadsheet program (Microsoft Office Excel 2019, Microsoft Corporation, Redmond, WA, USA) and a descriptive analysis was applied.

A statistical analysis of the results was performed using commercially available software SPSS V.21 for Mac. (SPSS, Chicago, IL, USA). To evaluate the effectiveness of diagnostic studies we assessed the sensitivity, specificity, diagnostic accuracy, positive, and negative prognostic values. k-statistics was used to quantify the degree of agreement and therefore to assess the interobserver variability. ROC-analysis (receiver operating characteristic) with AUC (area under the curve) evaluation was made for each observer on the binary scale of suspicion (EPNI yes/no). The survival analysis was made using Caplan Meier curve with cox regression analysis for evaluation of hazard ratios.

Data are presented as mean with standard deviation (SD). The significance level for statistical testing was set at $p<$ 0.05.
